# The crosstalk between lncRNA and microRNA in cancer metastasis: orchestrating the epithelial-mesenchymal plasticity

**DOI:** 10.18632/oncotarget.13957

**Published:** 2016-12-15

**Authors:** Ming-xin Cao, Ya-ping Jiang, Ya-ling Tang, Xin-hua Liang

**Affiliations:** ^1^ State Key Laboratory of Oral Diseases, West China Hospital of Stomatology (Sichuan University), Chengdu Sichuan, Peoples Republic of China; ^2^ Department of Oral and Maxillofacial Surgery, West China Hospital of Stomatology (Sichuan University), Chengdu Sichuan, Peoples Republic of China; ^3^ Department of Implant, The Affiliated Hospital of Qingdao University, Qingdao,Shandong, Peoples Republic of China; ^4^ Department of Oral Pathology, West China Hospital of Stomatology (Sichuan University), Chengdu Sichuan, Peoples Republic of China

**Keywords:** lncRNA, microRNA, epithelial-mesenchymal plasticity, cancer metastasis

## Abstract

Noncoding RNAs (ncRNAs) have been demonstrated to closely associate with gene regulation and encompass the well-known microRNAs (miRNAs), as well as the most recently acknowledged long noncoding RNAs (lncRNAs). Current evidence indicates that lncRNAs can interact with miRNAs and these interactions play crucial roles in cancer metastasis, through regulating critical events especially the epithelial-mesenchymal transition (EMT). This review summarizes the types of lncRNA-miRNA crosstalk identified to-date and discusses their influence on the epithelial-mesenchymal plasticity and clinical metastatic implication.

## INTRODUCTION

**Figure 1:**
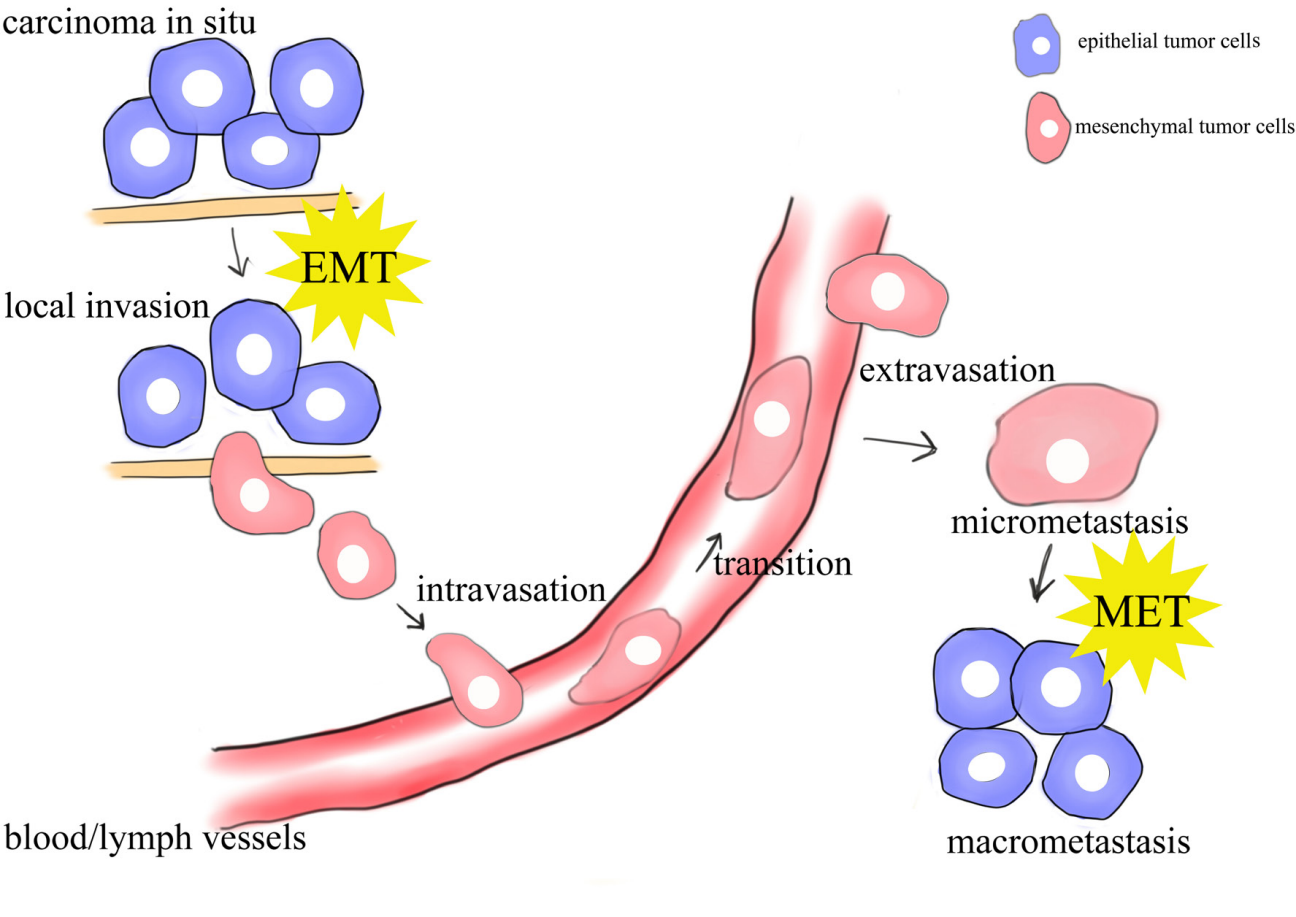
schematic mode of the sites of EMT/MET in the metastasis of cancer Epithelial cells undergo epigenetic changes and genetic alterations to become carcinoma *in situ*. Further alterations can induce local invasion of tumor cells, possibly through an EMT. The cells can intravasate into nearby blood and lymphatic vessels, be transported through the systems, and extravasate into the parenchyma of distant tissues. These cells may either be solitary or form a secondary tumor through a MET.

Cancer stems from the introduction of genetic mutations in normal cells to the biochemical changes of chromatin, signaling pathways and cell biological processes [1, 2]. The final manifestation of this disease usually comes to metastasis, responsible for more than 90% of cancer-associated mortality [3]. Many experimental and clinical studies have been tried to underlie the biology of this metastatic cascade. And there comes epithelial-mesenchymal plasticity, involving “changing faces” between epithelial cells and mesenchymal cells [4, 5]. Epithelial cells can undergo multiple biochemical changes to get a mesenchymal cell phenotype (EMT), and its reversible process, mesenchymal-epithelial transition (MET), can revert the mesenchymal cells back to epithelial cells [6, 7]. This bidirectional process contributes to the invasion-metastasis cascade from local invasion, then intravasation into nearby blood and lymphatic vessels, transition through the systems, extravasation into the parenchyma of distant tissues, finally colonization at particular distant sites [8, 9] (Figure 1). Given that emerging evidence has supported that de-differentiation of cancer cells through EMT with enhanced motility and dissemination, and re-differentiation through MET with colonization are critical in the course of multi-stage tumor progression, targeting EMT plasticity is thought to be a promising way to treat metastasis [10, 11]. Therefore, understanding the molecular mechanisms of governing EMT/MET in cancer metastasis cascade is vitally important.

Noncoding RNAs (ncRNAs), though do not encode proteins, contain genetic information or have function in the biological process of cells. NcRNAs include structural RNAs such as rRNAs and tRNAs involved in mRNA translation, small nuclear RNAs (snRNAs) involved in splicing, and regulatory RNAs such as microRNAs (miRNAs) and long noncoding RNAs (lncRNAs) [12]. All of them have important roles in regulating gene expression in development, physiology and pathology. Among these ncRNAs, the well-known miRNAs (~22nts), which are considered as central post-transcriptional gene regulators through their complementarity with the target mRNA sequences [13], and lncRNAs ( > 200nts), known as the “transcriptional noise”, which exhibit numerous functions in normal and abnormal tissues, are developing gradually [14, 15]. Recently, there is an interesting cross-regulation between lncRNAs and miRNAs, and the emerging evidence provides that this crosstalk has a great impact on the mechanisms of cancer metastasis [16]. In this review, we summarized miRNAs’ and lncRNAs’ control of EMT/MET, emphasized the influence of lncRNA-miRNA crosstalk in this multi-step process of human tumor progression, and harnessed this knowledge for translational medicine.

## KEY REGULATORS OF EMT/MET

Key regulators of EMT were categorized into three groups, including EMT effectors, EMT core regulators, and EMT inducers [1]. EMT effectors usually are proteins, which demarcate the epithelial or mesenchymal identity of a cell such as E-cadherin, α-catenin, γ-catenin and vimentin or promote cell migration and invasion during EMT like fibronectin. Decreased E-cadherin, α-catenin, γ-catenin and increased vimentin, fibronectin are consistent markers during EMT. Among them, E-cadherin is regarded as the leading one [17]. EMT core regulators consist of transcription factors, including Snail1 and Snail2, Zeb1 and Zeb2, Twist1 and Twist2, and the newly discovered paired-related homeobox transcription factor 1 (Prrx1) [5, 17-20], which dynamically modulate EMT mainly by regulating the expression of E-cadherin [21, 22]. EMT inducers are many signaling pathways, including TGF-β, Wnt, Notch and growth factor receptor signaling cascades [23, 24]. Most notably, the TGF-β pathway appears to be a primary inducer of EMT [6, 25, 26]. Besides, tumor inflammation and hypoxia microenvironment also play fundamental roles in promoting EMT [27, 28].

Conversely, MET is characterized by decreased expression of mesenchymal markers, such as N-cadherin and vimentin, and concomitant increased expression of epithelial markers, such as E-cadherin and CK-19 [6]. Recent studies have pointed out that BMPs, as multifunctional cytokines of the TGF-β superfamily, have been involved in mediating MET programs and boosting metastatic outgrowth by antagonizing the activities of TGF-β [7].

Recently, EMT is reported to be regulated by post-transcription factors, such as miRNAs and lncRNAs, which exert their influence by regulating effectors, transcription factors and signaling pathways [29, 30]. The underlying molecular mechanisms of EMT/MET has refined our understanding of how this phenomenon may be affected by post-transcription factors like miRNAs and lncRNAs.

## MICRORNAS’ CONTROL OF EPITHELIAL-MESENCHYMAL PLASTICITY

MiRNAs exert their influences, most of which are repressive, through targeting not only mRNAs, but also DNA and proteins [31]. Given that EMT can be regulated by post-transcription factors, a field of study has emerged requiring more efforts on the miRNAs control of EMT. Recently, a link between miRNAs and EMT has been extensively elucidated, and numerous miRNAs have been discovered to impact the process of EMT [32-35].

### EMT-inhibiting miRNAs

MiR-200 family members, including miR-200a, b, c, miR-141 and miR-429, are the first discovered, and also the most widely studied EMT-related miRNAs [36-38]. Decreased expressions of them suppress E-cadherin and initiate EMT by targeting transcription factors ZEB1 and ZEB2. Conversely, ectopic expressions of these miRNAs in mesenchymal cells induce MET through downregulating ZEB1/2 levels, and thus increasing E-cadherin and decreasing N-cadherin [39-41]. Given the fact that miR-200 family members inhibit expression of ZEB by binding to highly conserved target sites in their 3’UTRs and ZEB factors in turn repress the genes of miR-200 family members by binding to highly conserved recognition sequences in their promoters, Emerging data have demonstrated that there is a double-negative feedback loop between them. This feedback loop controls EMT/MET process through balanced expression of miR-200 family and ZEB factors in cancer microenvironment [42]. Further studies find more evidence about the miR-200 family. miR-200c inhibits metastasis of breast cancer by downregulating high mobility group protein B1 (HMGB1), which enhances tumor cell motility and suppresses apoptosis [43]. The miR-200 family and the miR-183~96~182 cluster which are co-repressed in lung cancer inhibit EMT and metastasis by inducing forkhead box F2 (Foxf2), which correlates with ZEB1, represses E-cadherin and forms a double-negative feedback loop with the miR-200 family [44]. Though double-negative feedback loop between the miR-200 family and ZEB1/2 remains to be the most widely existed in many types of cancer [45, 46], it has also been applied in many other miRNAs such as miR-203 and miR-145. In breast cancer, Snail2 reduces expression of miR-203, while ectopic expression of miR-203 directly represses Snail2. The Snail2 and miR-203 regulatory loop is in concert with miR-200 and ZEB1/2, forming a feed-forward loop to regulate EMT and gene expression [47]. Similarly, the mutual control of miR-145 and ZEB2 contributes to prostate cancer progression and metastasis, wherein ZEB2 directly represses the transcription of miR-145, which in turn represses ZEB2 [48]. These reciprocal feedback loops may explain the reversibility of EMT and MET through an imbalanced expression of miRNAs and EMT transcription factors [49].

In addition to forming feedback loops, miRNAs can directly target transcription factors or signaling pathways of EMT. MiR-106b inhibits EMT and metastasis of endometrial cancer *in vitro* by directly downregulating Twist1 mRNA at the 3’UTR [50]. Reduced expression of miR-145 promotes lung cancer cell EMT and metastasis *via* targeting octamer-binding transcription factor 4 (Oct4) mediated Wnt/β-catenin signaling pathway [51]. Additionally, miRNAs also affect the integrity of the epithelial and mesenchymal architecture, thus regulating EMT. In gastric cancer cells, miR-30a directly targets the 3’UTR of vimentin, inhibits its protein level, thus decreasing EMT and cell invasion [52]. The expression of miR-30a is increased by overexpression of a putative tumor suppressor, Runt-related transcription factor 3 (RUNX3) [53]. MiR-506 suppresses EMT and metastasis of ovarian cancer through the direct downregulation of two mesenchymal marker proteins, vimentin and N-cadherin *in vitro* and *in vivo* [54]. Another EMT-inhibiting miRNA is miR-138, which suppresses EMT in head and neck squamous cell carcinoma cell lines *via* three distinct pathways: (a) by regulating the expression of vimentin, (b) by targeting ZEB2, (c) by the epigenetic regulator enhancer of zeste homolog (EZH2) [55, 56]. Except for the miRNAs mentioned above, many new scenes have taken on in this field. Stromal interaction molecule 1 (STIM1), an endoplasmic reticulum Ca^2+^ sensor, is regulated by a post-transcriptional regulatory mechanism mediated by a novel EMT-inhibiting miRNA named miR-185 in the metastasis cascade of colorectal cancer [57].

### EMT-activating miRNAs

**Figure 2:**
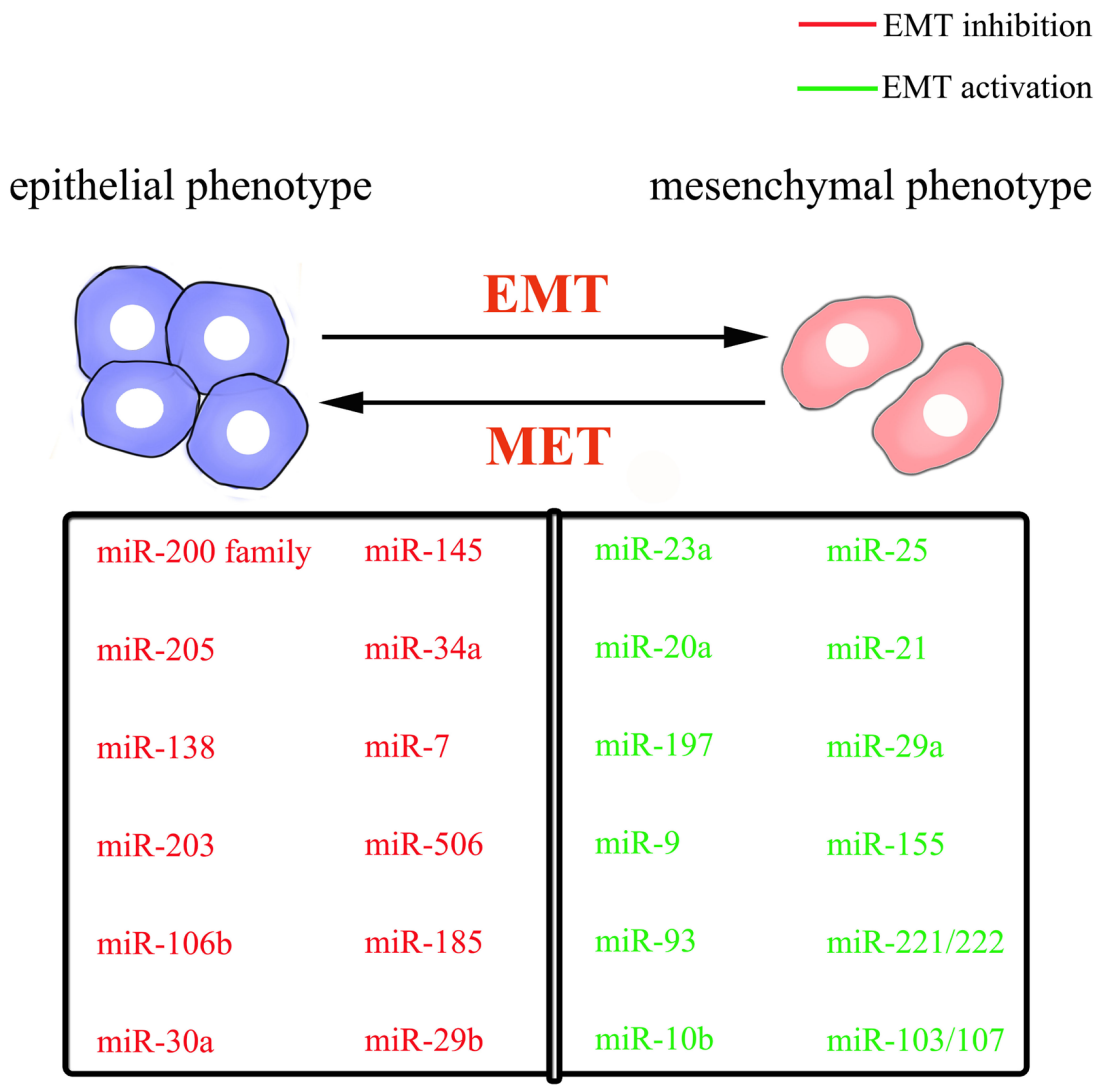
schematic mode of epithelial-mesenchymal plasticity and selected miRNAs important for the epithelial or mesenchymal phenotype

Compared with those miRNAs that suppress the EMT process, numbers of EMT-promoting miRNAs are relatively low. MiR-10b, the first reported metastasis-related miRNA, could trigger the EMT of laryngeal carcinoma by directly targeting E-cadherin mRNA [58, 59]. E-cadherin is also targeted by miR-9 and miR-23a. MiR-9, which is frequently overexpressed in esophageal squamous cell carcinoma, promotes metastasis by directly targeting E-cadherin and increasing β-catenin nuclear translocation, and subsequently inducing EMT [60]. Through TGF-β/Smad pathway [61], MiR-23a directly targets E-cadherin, promoting the mesenchymal phenotype with increased cell migration and invasion [62]. MiR-197 acts as an inducer of EMT in pancreatic cancer cells by indirectly targeting E-cadherin and regulating its membrane localization and trafficking *via* p120 catenin, an E-cadherin interaction protein [63]. Moreover, EMT-promoting miR-25 has been shown to be activated by the Wnt/β-catenin signaling pathway. Upregulation of miR-25 can inhibit the Rho GDP dissociation inhibitor alpha (RhoGDI1), enhancing expression of Snail and exerting its pro-metastatic function in hepatocellular carcinoma [64]. TGF-β1-induced miR-20a directly inhibits Smad7, thus enhancing the activity of β-catenin pathway and inducing EMT in gallbladder carcinoma [65]. Another refers to miR-93, which results in the attenuation of Smad-dependent TGF-β signaling and the activation of PI3K/Akt pathway by suppressing TGFBR2, promoting nasopharyngeal carcinoma cell uncontrolled metastasis and EMT-like process [66]. According to Lamouille's report, TGFBR2, also targeted by miR-302/miR-372 family and miR-106 family, plays a key role in regulating MET and maintaining the mesenchymal state [49]. In conclusion, these miRNAs profiles in the regulation of EMT/MET programs have developed gradually and become a tool to understand cancer metastasis (Figure 2).

## LNCRNAS’ CONTROL OF EPITHELIAL-MESENCHYMAL PLASTICITY

Although the functional roles of miRNAs in cancer metastasis are now well established, comparatively less is known about the regulatory roles of lncRNAs and their relevance to human diseases [67, 68]. Most of lncRNAs do not code for proteins, but interact with them [69, 70]. They are regulated like that of coding RNAs, subjecting to transcriptional regulation or even splicing, processing at the 5’ and 3’ ends, and exporting to the cytoplasm [71, 72]. Unlike microRNAs acting mainly as post-transcriptional repressors, Functional lncRNAs influence EMT in cancer metastasis through regulating gene expression at various levels, including chromatin modification, transcriptional and post-transcriptional processing [73, 74].

As the first disease-related ncRNA, MALAT-1, induced by TGF-β in cancer cells, promotes EMT by interacting with suppressor of zeste 12 (suz12), the subunit of polycomb repressive complex 2 (PRC2), then leading to E-cadherin downregulation [75, 76]. Suz12 functions as a histone H3 lysine 27 (H3K27) methyltransferase to bind E-cadherin promoter and suppress its expression in a PRC2-dependent way [77]. Besides chromatin modification, MALAT-1 also functions as transcription regulator through activating Wnt signaling pathway, which results in increasing of ZEB1, ZEB2, Snail2 and decreasing of E-cadherin in bladder cancer, or suppressing PI3K-AKT pathway and inhibiting EMT in breast cancer [78, 79]. Kan et al demonstrated that the expression of MALAT-1 can be enhanced by TADC-derived CCL5 in tumor microenvironment, subsequently increasing Snail expression [80]. Additionally, Shen et al found that silencing MALAT-1 may induce MET in the highly invasive subline of brain metastasis lung cancer cells, though the underlying mechanisms were not fully understood [81]. There are other lncRNAs functioning by chromatin modification, such as HOTAIR and H19. Both of them have been reported to interact with enhancer of zeste homolog 2 (EZH2), which also functions as a H3K27 methyltransferase when part of PRC2, to epigenetically inhibit genes responsible for suppressing cancer development, and increase metastasis [82-85].

LncRNAs perform their functions by regulating transcription through a broad spectrum of mechanisms. Linc00617 stimulates EMT *via* activating the transcription of Sox2 which promotes the oncogenic activity of breast cancer cells [86]. Wang demonstrated that lncRNA AOC4P inhibited EMT by binding vimentin and promoting its degradation in hepatocellular carcinoma [87]. Additionally, it was recently reported that some lncRNAs can be influenced by tumor microenvironment. An aberrant IL-6/STAT3/lncTCF7 signaling axis, in which IL-6 in tumor microenvironment induces lncTCF7 *via* activating STAT3, contributes to hapatocellular carcinoma cells aggressiveness through EMT induction [88]. Another example is a liver metastasis specific lncRNA, HULC, which can also be affected by liver micro-environment [89].

Furthermore, several lncRNAs can serve as antisense transcripts forming duplex with their corresponding mRNA counterparts to either induce or inhibit their translation, and thus influencing EMT. ZEB1-AS1 can induce EMT through positively regulating ZEB1 expression [90]. Ectopic overexpression of ZEB2NAT, which was founded in bladder cancer recently, prevents splicing of the *ZEB2* 5’-UTR, increases the levels of Zeb2 protein, and consequently downregulates E-cadherin mRNA and protein [91]. Further examples of antisense transcripts include 91H, ARNL (CDKN2B-AS1), and HNF1A-AS1 [92, 93].

In conclusion, Table 1 lists several selected lncRNAs with established roles in the EMT process. These representative lncRNAs are selected to illustrate the diverse mechanisms in EMT (Table 1).

**Table 1:** selected EMT-related lncRNAs showing diverse mechanisms of action

LncRNA	Mechanisms of action	References
**MALAT-1**	Interacts with suz12 resulting in suppression of E-cadherin and activation of N-cadherin and fibronectin. Affected by tumor micro-environment.	Ji et al. [75] Fan et al. [76] Kan et al. [80]
**HOTAIR**	Reprograms chromatin state.	Qiu et al. [82] Wu et al. [83] Kim et al. [84]
**BANCR**	Histone de-acetylation suppresses BANCR to promote EMT.	Guo et al. [96] Sun et al. [97]
**H19**	Chromatin modifier.	Matouk et al. [85]
**Linc00617**	Stimulates EMT via activating the transcription of Sox2.	Li et al. [86]
**LncRNA-HIT**	Promotes TGF-β-induced EMT via downregulating the levels of E-cadherin.	Richards et al. [98]
**AOC4P**	Inhibits EMT via binding to vimentin and promoting its degradation.	Wang et al. [87]
**HULC**	Affected by tumor micro-environment.	Matouk et al. [89]
**LncTCF7**	Affected by tumor micro-environment.	Hamada et al. [63]
**ZEB1-AS1**	Upstream antisense RNA enhances ZEB1 expression.	Li et al. [90]
**Zeb2NAT**	Prevents splicing of the Zeb2 5’-UTR, increases Zeb2 and downregulates E-cadherin.	Zhuang et al. [91]
**91H**	H19 antisense. Associated with H19 ICR methylation.	Deng et al. [92] Gao et al. [93]

Abbreviations: AOC4P, amine oxidase, copper containing 4, pseudogene; BANCR, BRAF-activated non-protein coding RNA; EMT, epithelial-mesenchymal transition; HOTAIR, HOX transcript antisense RNA; HULC, highly up-regulated in liver cancer; lncRNA, long noncoding RNA; MALAT-1, metastasis-associated lung adenocarcinoma transcript 1; Sox2, SRY (sex determining region Y)-box 2; TGF-β, transforming growth factor beta; ZEB1/2, zinc-finger E-box-binding homeobox 1/2.

## LNCRNA-MICRORNA INTERACTIONS’ CONTROL OF EPITHELIAL-MESENCHYMAL PLASTICITY

While the action of miRNAs and lncRNAs as controllers of EMT/MET in cancer metastasis has been discussed, a number of new findings over the past decade have begun to uncover the interactions between lncRNAs and miRNAs in this process [94, 95, 99]. In some cases, lncRNA stability will be reduced due to the interaction with specific miRNAs. In other cases, lncRNAs, also known as competing endogenous (ce)RNAs, can sequester miRNAs away from their target mRNAs by binding miRNAs, therefore antagonizing miRNAs [100]. LncRNAs can also compete with miRNAs by binding mRNAs. What's more, some lncRNAs can produce miRNAs, causing repression of target mRNAs. These studies suggested that interplay patterns between lncRNAs and miRNAs may have an impact on cancer development and progression, so it is necessary to further dig into these interactions in the biological process of cancer [95]. The lncRNA-miRNA interactions identified to-date are summarized in Table 2.

**Table 2:** EMT-related lncRNA-miRNA interactions identified to-date

LncRNA	MicroRNA	Mechanisms of interaction	Cancer types	References
**MALAT-1**	miR-217	miR-217-triggered MALAT-1 decay	lung cancer	Lu et al. [101]
	miR-9	miR-9-triggered MALAT-1 decay	osteosarcoma	Fang et al. [102]
	miR-1	reciprocal negative control between MALAT-1 and miR-1	breast cancer	Jin et al. [103]
**HOTAIR**	miR-7	HOTAIR downregulates miR-7 by inhibiting HoxD10	breast cancer	Zhang et al. [114]
	miR-568	HOTAIR downregulates miR-568 by chromatin modification	breast cancer	Li et al. [115]
**lncRNA-ATB**	miR-200s	lncRNA-ATB acts as sponge of miR-200s	hepatocellular and gastric cancer	Yuan et al. [104] Saito et al. [105]
**H19**	let-7	H19 acts as sponge of let-7	pancreatic cancer	Ma et al. [106]
	miR-138 miR-200a	H19 acts as sponge of miR-138 and miR-200a	colorectal cancer	Liang et al. [107]
	miR-141	H19 acts as sponge of miR-141	gastric cancer	Zhou et al. [108]
	miR-675	H19 generates miR-675	prostate cancer	Zhu et al. [112]
**lincRNA-ROR**	miR-205	lincRNA-ROR acts as sponge of miR-205	breast cancer	Hou et al. [109]
**ZFAS1**	miR-150	ZFAS1 acts as sponge of miR-150	hepatocellular carcinoma	Li et al. [110]
**UCA1**	miR-145	reciprocal negative control between UCA1 and miR-145	bladder cancer	Xue et al. [111]

Abbreviations: EMT, epithelial-mesenchymal transition; HOTAIR, HOX transcript antisense RNA; HoxD10, homeobox D10; lncRNA, long noncoding RNA; MALAT-1, metastasis-associated lung adenocarcinoma transcript 1; miRNA, microRNA; UCA1, the urothelial cancer associated 1.

### miRNAs triggering lncRNAs to decay

MiR-217, a tumor suppressor, can inhibit MALAT-1 through the Ago2-mediated pathway, and thus inhibit EMT by suppressing EZH2-mediated H3K27me3, upregulating E-cadherin and downregulating N-cadherin and vimentin in cigarette smoke extract (CSE)-induced malignant transformation of HBE cells [101]. Similarly, the recruitment of miR-9 by 17β-Estradiol also causes decreased stability of MALAT-1 in osteosarcoma cell MG-63, and inhibits migration and invasion [102].

### lncRNAs binding miRNAs to derepress mRNAs

There are lncRNAs which harbor similar miRNA target sequences, acting as miRNA sponges and hence sequestering miRNAs away from mRNAs, thereby derepressing mRNAs. For example, MALAT-1, which has complementary base pairing with miR-1, upregulates Slug expression through inhibiting miR-1 expression, and thus promotes EMT in triple-negative breast cancer [103]. Another was reported for lncRNA-ATB, which upregulated ZEB1 and ZEB2 by competitively binding the miR-200 family, and then induced EMT and invasion in both hepatocellular carcinoma and gastric cancer [104, 105]. Ma proposed that H19 promoted pancreatic ductal adenocarcinoma (PDAC) cell invasion and migration at least partially through antagonizing let-7 and then leading to derepression of its target high mobility group protein A2 (HMGA2) [106]. Additionally, other researches show that H19 also can act as ceRNAs for miR-138, miR-200a and miR-141 in different cancer types respectively. Liang demonstrated that H19 can antagonize functions of miR-138 and miR-200a and led to the derepression of their endogenous targets vimentin, ZEB1 and ZEB2, thus promoting EMT in colorectal cancer [107]. With regard to miR-141, Zhou was the first to demonstrate that H19 and miR-141 could compete with each other and affect their target genes in gastric cancer [108]. Other examples are involved in lincRNA-ROR, ZFAS1 and lncRNA-UCA1. These lncRNAs can act as ceRNAs and contribute to EMT and cancer metastasis [109-111].

### lncRNAs generating miRNAs

LncRNAs are also processed to generate miRNAs. Zhu found that H19 could generate miR-675 which suppressed prostate cancer EMT and metastasis by downregulating transforming growth factor β induced protein (TGFBI), an extracellular matrix protein involved in cancer1metastasis [112]. This process during which H19 generates miR-675, is repressed by stress-response RNA binding protein HuR in a Drosha-dependent manner and dynamically regulated *in vivo* [113]. In addition, lncRNA HOTAIR, which is highly expressed in metastatic breast cancers, accelerates the EMT-dependent metastasis of breast cancer by inhibiting miR-7 through HoxD10 inhibition [114]. HOTAIR also transcriptionally inhibits the expression of miR-568 by directly targeting NFAT5 that promotes EMT in breast cancer [115]. However, further investigations are warranted to elucidate the relationship between lncRNAs and miRNAs for better therapeutic strategies.

## SUMMARY AND FUTURE PERSPECTIVES

LncRNAs and miRNAs regulate gene expression involved in epithelial-mesenchymal plasticity on all levels. Through this sophisticated and multi-layered influence on protein expression patterns, these noncoding RNAs affect cancer metastasis and prognosis. Here, we have summarized the examples of a newly-developing mechanism - crosstalk between lncRNA and miRNA, and its influence on cancer metastasis cascade.

This crosstalk points out a novel way to understand the RNA networks. For example, the way miRNAs reduce lncRNA stability as described before is not entirely unexpected, since lncRNAs resemble mRNAs in many aspects. But whether or not miRNAs also regulate the transcription or splicing of lncRNAs requires further study [116, 117]. In addition, some lncRNAs have the ability of sequestering a handful of miRNAs as a ceRNA just like H19, and one miRNA can also control many genes, thus making this crosstalk more complicated [85]. Apart from the direct interactions mentioned before, there may exist some indirect actions. For example, lacking an mRNA specific target site, miRNA can transcriptionally repress mRNA through lowering the levels of a miRNA-related lncRNA. Another challenge to hamper the application of the crosstalk mechanism in clinic is which cellular conditions should exist for the network to work, since researches evidenced that the relative concentration of lncRNAs and miRNAs must be suitable for interaction [118, 119]. Moreover, EMT/MET, as a complex bidirectional process, seems difficult to target. Inhibiting EMT or blocking cancer cell invasion may be applicable in early stage carcinomas. But once cancer cells have disseminated from the primary site, inhibiting EMT may be counterproductive, since it is beneficial for MET [120]. So finding out the exact mechanism in the secondary site colonization is quite important.

In conclusion, lncRNA-miRNA crosstalk not only suggests the existence of a complex regulatory network in cancer, but also implies the possibility of cancer diagnosis and therapy using this panel of network. Though in its infancy, its ability to contribute to cancer metastasis is continually being validated.
